# Discrete Event System Specification for IoT Applications

**DOI:** 10.3390/s24237784

**Published:** 2024-12-05

**Authors:** Iman Alavi Fazel, Gabriel Wainer

**Affiliations:** Systems and Computer Engineering, Carleton University, Ottawa, ON K1S 5B6, Canada; gwainer@sce.carleton.ca

**Keywords:** IoT, sensor fusion, DEVS, M&S

## Abstract

The Internet of Things (IoT) has emerged as a transformative technology with a variety of applications across various industries. However, the development of IoT systems is hindered by challenges such as interoperability, system complexity, and the need for streamlined development and maintenance processes. In this study, we introduce a robust architecture grounded in discrete event system specification (DEVS) as a model-driven development solution to overcome these obstacles. Our proposed architecture utilizes the publish/subscribe paradigm, and it also adds to the robustness of the proposed solution with the incorporation of the Brooks–Iyengar algorithm to enhance fault tolerance against unreliable sensor readings. We detail the DEVS specification that is used to define this architecture and validate its effectiveness through a detailed home automation case study that integrates multiple sensors and actuators.

## 1. Introduction

The Internet of Things (IoT) refers to a network of embedded devices that interact with the environment through sensors and actuators for the purpose of monitoring and controlling environmental variables. These systems integrate processing units and use various technologies for communication, including both wired and wireless. A common application of IoT is in home automation, where sensors and actuators enable precise control of heating, ventilation, and air conditioning (HVAC) systems. This setup allows for the automation of routine tasks and enhances energy efficiency. Beyond home automation, IoT has been applied to various domains, including transportation and healthcare. In transportation, IoT technologies enhance vehicle tracking, whereas in healthcare, they facilitate remote monitoring and the use of advanced medical devices, improving patient care and operational efficiency.

While IoT has proven to be effective in various domains, implementing and validating IoT systems comes with specific challenges. These include interoperability, heterogeneity, and the “human-in-the-loop” concept. Interoperability refers to the capacity of different system components to operate and communicate effectively with one another. Heterogeneity involves the integration of diverse technologies within systems. Lastly, “human-in-the-loop” signifies that human involvement is essential for the system’s operation.

To overcome the challenges associated with developing IoT systems, model-based approaches have been proposed in the literature. These techniques involve using models expressed in various formats, such as textual or graphics, to build applications with high-level semantics. The use of high-level semantics provides abstraction, separation of concerns, and reusability [1,2].

In particular, discrete event system specification (DEVS) is a modeling formalism that has been applied to the model-driven development of embedded real-time systems consisting of sensors and actuators [3]. DEVS extends the concept of finite-state machines (FSMs) by allowing state transitions to occur by both new inputs and the expiration of a time variable. By adopting DEVS, a series of models can be developed and used for both simulation and deployment on the hardware. This is achieved by first using a DEVS simulator to verify the correctness of the models prior to their deployment and then flashing the models alongside the DEVS simulator onto the hardware.

Other advantages of DEVS include its hierarchical and modular nature. These characteristics enable the implementation and verification of smaller models, which can then be coupled to form a complete model of interest. In this process, models can be reused and integrated with multiple other models. Lastly, DEVS allows for the simulation of these models alongside a set of well-developed models, targeting both discrete-event and continuous systems [4].

Apart from the challenges mentioned in the development of IoT systems, a recurring issue is the potential “single point of failure” of individual sensors. This occurs when a sensor becomes faulty due to factors such as the harsh environment in which it is deployed. If such systems rely on a single sensor for their operation, the failure of the sensor results in incorrect readings propagating through the system, which can cause the actuators to perform incorrect actions on the environment.

To mitigate single points of failure, a network of redundant nodes can be deployed in the environment, with each node equipped with one or more redundant sensors. Such a setup requires the use of a suitable algorithm to handle the added redundancy in the system.

One approach that can be used for this purpose is the Brooks–Iyengar algorithm. This algorithm allows the nodes to combine readings from other nodes into values that are free from faulty readings. Additionally, the nodes can exchange multiple rounds of messages and, after reapplying the algorithm, reach an agreement on certain values.

The objective of this work is to employ DEVS model-based architecture to design and develop IoT applications and to integrate models implementing the Brooks–Iyengar algorithm as part of this methodology to achieve improved fault tolerance in these systems. The Brooks–Iyengar algorithm was chosen for its effectiveness in handling a network of redundant sensors, such as in an IoT setup, where the sensors poll out of phase with each other and network delays are present.

The main contribution of the work includes defining a set of DEVS models for IoT nodes, including one that implements the Brooks–Iyengar algorithm. Additionally, the methodology was developed for nodes communicating within a publish/subscribe architecture, a de facto standard in many IoT applications.

The developed models on the sensor nodes achieve sensor fusion and agreement by dividing the environment into discrete “zones”. For each zone, a separate publish/subscribe “topic” was defined. Each node then published to and subscribed to the topic associated with its zone. Consequently, all nodes within a zone communicated with one another.

As a case study, a home automation application was developed. This application consisted of multiple nodes for both sensing the environment and controlling actuators that interact with it. The sensors included temperature and CO_2_ sensors. The actuators were a DC motor for heating, ventilation, and air conditioning (HVAC) and a servomotor that adjusts the level of a blind to allow varying degrees of light to enter the room.

The role of the nodes with sensors, or “sensor nodes”, was to sense the environment and, after reaching an “agreement” among themselves, transmit the readings to the node with the DC motor, referred to as the “HVAC node”.

The HVAC node received the sensor readings and controlled the DC motor based on these readings and the PID control principle. The PID control technique ensured that the motor’s speed was adjusted precisely to achieve the desired temperature and CO_2_ levels within the specified time while maintaining smooth operation. The node with the servomotor was controlled independently of sensor readings and relied solely on user input. Together, these nodes constituted a complete IoT setup for a home automation application. The models were simulated using a DEVS simulator, and after verifying their correct behavior, they were flashed onto microcontrollers along with the DEVS simulator. Finally, these nodes were installed in a miniature room model and tested in real time.

The rest of the work is organized as follows: Section 2 provides background on IoT, discusses prior work on model-driven development and DEVS, and introduces fault-tolerant techniques used for IoT. Section 3 details the main architecture developed in this work, including the description and specification of the DEVS models. Section 4 presents a case study on home automation. Lastly, Section 5 concludes the work and outlines future directions for this research.

## 2. Related Work

Various approaches in the literature leverage high-level models for the design of IoT applications. These techniques, known as model-driven development (MDD), enable the design of IoT devices using high-level semantics rather than platform-specific programs. The models can be expressed in various formats, such as graphical or textual, with either formal or informal semantics. However, their common goal is to create an abstraction over specific code implementations. After their design and analysis, these models will eventually be transformed into executable code that can be run on the hardware. In MDD, the use of high-level models provides abstraction, separation of concern, and reusability to the development cycle [1,2]. The survey [5] outlines the previous works in the literature that used modeling language for IoT development. The survey reveals that researchers employed various types of models for developing IoT applications, ranging from those based on visual notations to textual formats using domain-specific languages (DSLs). These models also were expressed with languages that followed either formal or informal semantics. Languages with formal semantics use a rigorous mathematical formalism that results in an unambiguous interpretation of the model. Examples of these languages are the ones that are based on Petri nets [6] and Temporal Logic [7]. On the other hand, models following informal semantics use expressions in plain English that can result in different interpretations of the same model. Other research developed tools to verify the models with techniques such as using simulation and model checking to ensure the correct behavior of the system. In what follows, some of these works are presented.

In [8], the authors presented a DSL that allows developers to define ports, properties, and state charts in a text-based format and later use a set of tools to automatically transform models and generate code. In [9], a UML-based approach was used to generate wrappers, enabling the integration of diverse IoT elements. The work [1] introduced a framework to enable node-centric and rule-based programming through a DSL, providing reusability, flexibility, and maintainability. The authors in [10] presented a method for designing and analyzing IoT applications to verify their correctness and QoS using SysML4IoT, a framework consisting of a SysML profile and a model-to-text translator that converts the models for a model checker. The authors in [3] applied the DEVS formalism in the context of embedded systems comprising sensors and actuators. As a case study, they later used their methodology to develop an automated manufacturing system (AMS).

In this work, we adopted the discrete event system specification (DEVS) formalism for the IoT application due to its capability for reusing models in both simulation and deployment. DEVS is a formalism that can be viewed as an extension of finite-state machines, where state transition occurs either due to new input to the system as well as the expiry of a time variable [4]. DEVS is modular and hierarchical, which means the models of interest are built from a set of “atomic models”, each with their own states and state transitions. The atomic models can then be coupled together to create more complex “coupled models”.

In IoT environments, a network of sensors operates as a distributed system, which introduces several challenges. Beyond interoperability and system complexity, a critical issue arises from faulty components that can generate inconsistent data, resulting in conflicting information being transmitted and received across different parts of the system. To address this issue and build systems that are resilient against this problem, researchers have formulated a problem known as the Byzantine Generals Problem [11]. A variant of the Byzantine Generals Problem, named “inexact agreement” was studied in [12]. The authors developed two algorithms for their problem formulation: namely, the Fast Convergence Algorithm (FCA) and the Crusaders Convergence Algorithm (CCA). In the inexact agreement problem, the nodes initially start with numerical real values that are within δ of the true value and ε of each other, where δ and ε are two constants. Then, their algorithms cause the values of the nodes to converge to a common by performing message exchanges between the nodes. Furthermore, convergence is guaranteed as long as less than one-third of the nodes are faulty. If the number of faulty nodes is between one-third and two-thirds, the algorithm either still converges but with less accuracy compared to the case where faulty nodes are fewer than one-third, or it stops executing. In the process of convergence, however, some nodes can deviate further from the true value of the environment. Both FCA and CCA achieve a similar goal, except that FCA converges faster, with the expense of an extra step in the algorithm. In their work, the authors used the FCA algorithm to propose a protocol that can be used for clock synchronization.

In the literature, another set of algorithms has been developed to achieve fault tolerance by combining multiple sensor readings together. These algorithms, referred to as “sensor fusion” algorithms, have a different focus compared to the “inexact agreement” problem, which aims solely at achieving consensus among distributed nodes. The survey [13] provides an overview of various sensor fusion approaches proposed in the literature that use techniques such as fuzzy logic, Bayesian inference, and Dempster–Shafer theory.

When dealing with replicated sensor readings, a particularly useful sensor fusion algorithm is proposed by Marzullo in [14]. In this algorithm, the sensor readings are in the form of intervals of values (rather than a single numerical value), and the output is also an interval that the true value of the environment is guaranteed to lie in. The output of this algorithm is an interval, which is the overlap of different readings greater than a threshold. In [14], a process control application that consists of a train that traverses track segments is also presented.

The Brooks–Iyengar algorithm [15] is a hybrid between the sensor fusion algorithm proposed by Marzullo and the FCA algorithm that was devised for an inexact agreement problem. Using this approach, the requirement of the inexact agreement can be achieved while the values that are computed are as close to the true environment’s variable as possible. In this algorithm, similar to Marzullo’s approach, the measurements are assumed to be intervals of values, and like FCA, the algorithm can be applied recursively to achieve the desired precision. Furthermore, the number of faulty nodes that the Brooks–Iyengar algorithm can tolerate is similar to FCA: one-third to guarantee convergence or between one-third and two-thirds with the possibility of either convergence or stopping execution. In this research, we integrated DEVS as a model-based approach and combined it with the Brooks–Iyengar algorithm to propose an innovative architecture designed specifically for IoT environments. This architecture aims to enhance system resilience by effectively managing and tolerating faulty readings from sensors, a common challenge in distributed IoT systems. By leveraging the formal modeling capabilities of DEVS, we can accurately simulate and analyze the behavior of the system under various conditions, while the Brooks–Iyengar algorithm facilitates robust data processing and error detection. This combination not only improves the reliability of data transmission but also ensures that the system can maintain operational integrity despite the presence of erroneous sensor inputs, ultimately leading to more dependable and efficient IoT solutions.

## 3. Brooks–Iyengar Algorithm in Publish/Subscribe Architecture

As discussed in the previous section, the Brooks–Iyengar algorithm can be used to achieve inexact agreement and sensor fusion in a distributed network of sensors. In this section, we describe an architecture to apply this algorithm in a network of devices that communicate using a publish/subscribe architecture and using the DEVS formalism.

**Figure 1:**
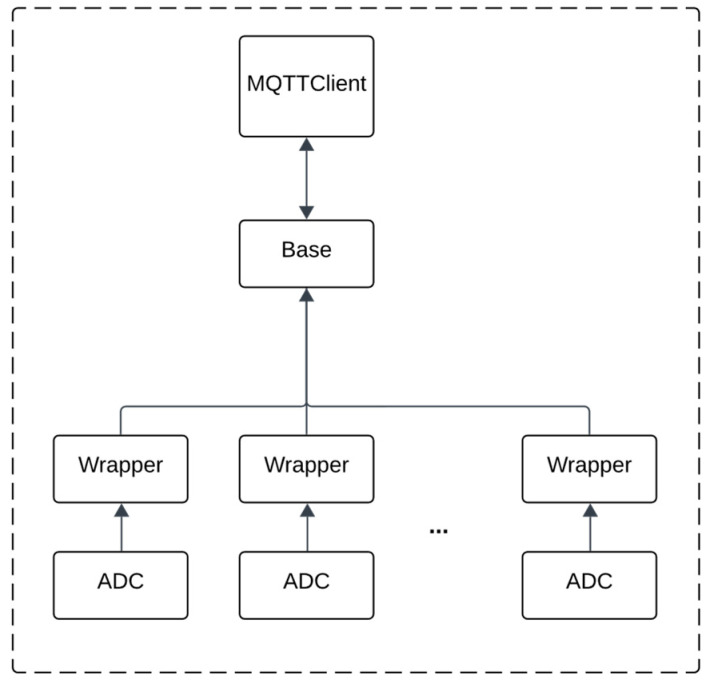
The coupled DEVS model developed to execute on IoT devices.

The DEVS atomic models defined are depicted in Figure 1. These models run on each device with a number of redundant sensors attached to them. The models are designed to read sensor data, apply the Brooks–Iyengar algorithm, and transmit data to other nodes.

**Figure 2:**
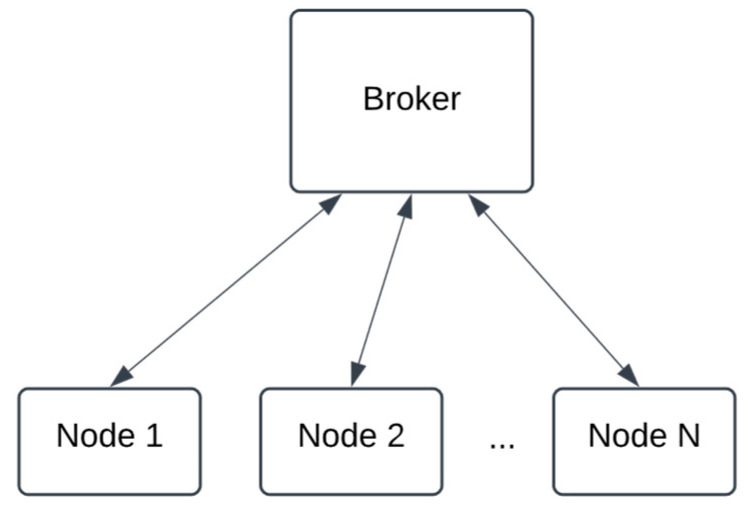
Multiple nodes communicating with a message broker.

**Figure 3:**
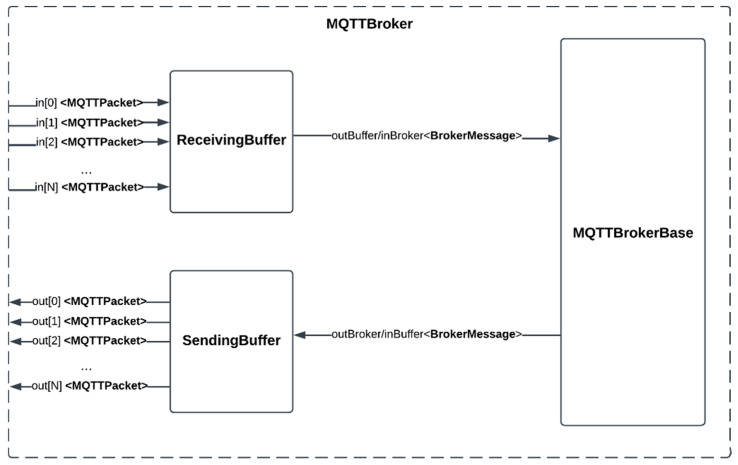
Input and output relations of the NetworkMedium model.

The DEVS models shown in Figure 2 operate on individual devices within the architecture, with multiple such devices deployed throughout the system. These nodes utilize the publish/subscribe paradigm for communication, facilitating efficient message exchange through a centralized message broker. This configuration is illustrated in Figure 3, highlighting the interconnectedness and collaborative functionality of the nodes within the proposed architecture.

The broker in Figure 2 was also modeled using DEVS to enable the simulation of communication among multiple nodes. For deployment, this DEVS model of the broker is substituted with a functional publish/subscribe message broker while the nodes continue to operate using the same models as demonstrated in the simulation. In this deployment configuration, the DEVS models within the nodes run on a DEVS simulator utilizing a real-time clock, as opposed to an event-based clock, ensuring seamless integration and real-time communication.

### 3.1. Overview of the DEVS Models Definitions

In Figure 1, the ADC models periodically poll the analog-to-digital channels of the devices and capture the values of their sensors. The captured value is the quantization of the analog signals into a digital number that corresponds to the voltage or the current level of the analog input. The quantization of the signal enables the devices to store sensor values in the memory registers and perform the desired computations on them. Each ADC model captures the value of one redundant sensor. Hence, depending on the number of redundant sensors, a variable number of ADC models may be used.

The ADC model component is formally defined using DEVS as the following atomic model (Model 1):
**Model 1** ADC ModelADC = <S, X, Y, δint, δext, λ, ta> S = {ADC_channels} X = {} Y = {ADC_output} δint (s) = { Polls ADC_channels and saves the data } λ () = {ADC_output = ADC_channels} ta () = pollingRate

The sensor values in the ADC models, after being captured via the internal transition function, are transmitted instantaneously through the output port of the model by the execution of the output function. In our architecture, the ADC models are connected to the Wrapper models. Each Wrapper model complements the sensor readings with additional information about the sensor’s origin and a feasible range of values. The information about their origin enables the subsequent models to distinguish the values of an ADC model from the other ADC models. For instance, if we consider an application in home automation, redundant sensors for both capturing CO_2_ levels as well as temperature might be deployed in the environment.

In these cases, the identifiers that are added by the Wrapper models enable the models that receive these values to perform different sets of computations depending on whether the received value is a temperature or CO_2_ level. Furthermore, the feasible range added by the Wrapper model gives information about the bound in which the actual value of the environment should be present. This range is needed to later apply the Brooks–Iyengar algorithm, as the input for this algorithm is a range of values. These ranges can be determined depending on the physical phenomenon being measured and the precision of the sensors. The Wrapper model is formally defined as Model 2:
**Model 2** Wrapper ModelWrapper = <S, X, Y, δint, δext, λ, ta> S = {phase ∈ {inputHasArrived, idle}, serializedInput} X = {adc_in} Y = {output} δext (s,e,x) = { phase = inputHasArrived serializedInput =      buildMessageObject(adc_in, FEASIBLE_RANGE, ORIGIN_OF_SENSOR) //FEASIBLE_RANGE and ORIGIN_OF_SENSORS are constants defined for the model } δint (s) = { phase = idle ta(phase) = Inf } λ (s) = {output = serializedInput} ta(phase) = 0

The advantage of having a separate model, i.e., the Wrapper model, to incorporate additional information is the ability to include the same set of data for multiple ADC models by connecting all of them to the same Wrapper model.

The outputs of multiple redundant sensors, after being processed by the Wrapper models, are forwarded to the Base model. Whenever the Base model receives values from all Wrapper models, it applies the Brooks–Iyengar algorithm on the sensor readings, hence performing “sensor fusion”, and sends the result to the MQTTClient model. The MQTTClient model then transmits these values through the network by publishing the data to a topic on an MQTT broker.

The Base model, in addition to sending the fused values to the MQTTClient, transmits a set of topics for the MQTTClient to subscribe to. Whenever the MQTTClient model receives value from its subscribed topics, it forwards them to the Base model through its output port. This allows the Base model to both send and receive fused values from other nodes. The Base model collects the received values from the other nodes and computes a new output using the Brooks–Iyengar algorithm. The result is then forwarded back to the MQTTClient to be sent to the neighboring nodes. Performing multiple rounds of message exchanges causes the nodes’ values to converge closer to each other, achieving “inexact agreement”.

In the Base model, a map is used to keep track of the values from neighboring nodes during the process of collecting these values and reapplying the algorithm. When this “map” becomes full, (i) the Base model applies the Brooks–Iyengar algorithm to the map and saves the result as the node’s current value. (ii) It clears the content of the map. (iii) The current value of the device is sent back to the MQTTClient for broadcast (in general, to the other nodes).

Additionally, the Base model increments an internal counter each time the map becomes full and is cleared. This counter corresponds to the number of messages the node sends to other nodes between each ADC polling. Once this counter reaches a limit, the model will discard any subsequent values received from neighboring nodes that are not added to the internal map. The choice of this counter limit determines the accuracy level of the node’s results; a larger limit allows values to converge more closely. For a comprehensive understanding of its convergence and analysis, please refer to the original work that introduced the algorithm [15].

The Base model is defined as Model 3:
**Model 3** Base ModelBase = <S, X, Y, δint, δext, λ, ta> S = {phase ∈ {active, passive}, dataToPublish, fusedInputValues, receivedInputMap,      counter} /* “dataToPublish” contains the result of the Brooks–Iyengar algorithm applied to       replicated sensors or neighboring nodes. It consists of two fields: - DATA: the fused sensor values. - ID: the unique identifier for each node. “receivedInputMap” is a map that associates each neighboring node with the value       it last reported during the convergence phase. */ X = {MQTTClient_IN, data_in} Y = {output} δext (s,e,x) = { if(data_in) then  dataToPublish = Brooks_Iyengar(data_in)  phase = active  counter = 0  dataToPublish.ID = SELF_ID   if(MQTTClient_IN) then   if(s.counter > COUNTER_LIMIT) then        //COUNTER_LIMIT is the desired max rounds of message exchange    phase = idle   return   receivedInputMap[MQTTClient_IN.ID] = MQTTClient.DATA   if(sizeof(receivedInputMap) > numberOfNeighboringNodes)   dataToPublish.DATA = Brooks_Iyengar(receivedInputMap)   dataToPublish.ID = SELF_ID    counter++   receivedInputMap.clear()   phase = active } δint (s) = { if(counter < COUNTER_LIMIT) then  phase = active  counter++ else phase = idle } λ (s) = {output = dataToPublish} ta(idle) = infinity ta(active) = MESSAGE_EXCHANGE_INTERVAL_RATE //A constant smaller than ADC polling rate that would ensure convergence between the      nodes between consecutive ADC polls

The MQTTClient is defined in a similar fashion.

To allow the simulation of the nodes’ communication with the publish/subscribe architecture, an additional DEVS model representing an MQTT Broker was developed. This model is a coupled model composed of three atomic models: the SendingBuffer, the ReceivingBuffer, and the MQTTBrokerBase. The SendingBuffer and ReceivingBuffer are queuing network models that mimic the latency of network transmission, while the MQTTBrokerBase models the actual message broker providing communication for MQTT clients connected to it. The MQTTBroker model is illustrated in Figure 3. As depicted, the model includes an array of input and output ports that send and receive data of type MQTTPacket to and from the ReceivingBuffer and SendingBuffer models, respectively. An object of type MQTTPacket contains the data sent by the client, the publish/subscribe topic of the message, and a flag indicating whether the message is “PUBLISH” or “SUBSCRIBE”.

The other ports in the SendingBuffer and ReceivingBuffer exchange messages of type BrokerMessage with the MQTTBrokerBase. An object of type BrokerMessage contains MQTTPacket objects, along with an integer value referring to the index of the buffer port where the packet either arrived at or is intended to be sent from within the MQTTBroker model. This index value helps the MQTTBrokerBase differentiate messages sent by different clients or broadcast to specific clients. Therefore, throughout the simulation, clients must remain connected to specific input and output ports of the model.

Internally, the buffer models append received data from their input ports to a queue. Packets in this queue are dispatched after a certain time interval, determined by an exponential distribution function.

The ReceivingBuffer and SendingBuffer share identical definitions except for the structure of their input and output ports. The ReceivingBuffer is defined as follows (Model 4):
**Model 4** ReceivingBuffer ModelReceivingBuffer = <S, X, Y, δint, δext, λ, ta> S = {phase ∈ {active, passive}, queue} X = {in[0], in[1], … in[N]}//Where N is the number of clients in the simulation Y = {outBroker} δext (s,e,x) = { for i in range(0, N)  if(in[i])   queue.add(in[i]) if(phase == passive) then  phase = active } δint (s) = { if(!queue.empty) then  queue.dequeue()   if(queue.empty) then    phase = passive } λ (s) = {output = queue.front} ta(active) = generate an exponentially distributed random number() ta(passive) = infinity

The SendingBuffer model has a similar definition, except that it has an array of input ports and a single output.

The MQTTBrokerBase receives MQTTPacket messages from clients, either of the type SUBSCRIBE or PUBLISH. Upon receiving a SUBSCRIBE message, it updates internal state variables that store information about the topics to which each client has previously subscribed. Conversely, when the model receives PUBLISH messages, it inspects the internal variables and, based on the clients subscribed to the topic, broadcasts the message to the appropriate clients.

The MQTTBrokerBase is defined as follows (Model 5):
**Model 5** MQTTBrokerBase ModelMQTTBrokerBase = <S, X, Y, δint, δext, λ, ta> S = {phase ∈ {passive, publishPacketArrived}, arrivedPacket, subscriptionMap} X = {inBroker} Y = {outBroker} δext (s,e,x) = { phase = passive ta(phase) = infinity } δint (s) = { if(inBroker) then  arrivedPacket = inBroker  if(arrivedPacket.type == SUBSCRIBE) then    subscriptionMap(arrivedPacket.topic).append(arrivedPacket.portNumber)   else if (arrivedPacket.type == PUBLISH) then    phase = publishPacketArrived } λ (s) = {  if(phase == publishPacketArrived)  tmp = []   for i in subscriptionMap[arrivedPacket.topic].length    tmp.append(BrokerMessage(subscriptionMap[arrivedPacket.topic][i],            arrivedPacket))    output = tmp } ta(publishPacketArrived) = 0 ta(passive) = infinity

### 3.2. Implementation of the Proposed Architecture

The DEVS models described in the previous section were implemented using the Cadmium library version 2. In Cadmium version 2, each atomic model is defined as a C++ class derived from the type Atomic<T>. Therefore, multiple C++ classes, alongside their state variable types, were defined to correspond to each model in our architecture. These models were used for simulation purposes and later flashed, alongside a DEVS simulator, onto hardware for deployment (The source code for the models is available at https://github.com/alavifazel/fault-tolerant-iot-devs, accessed on 26 November 2024). All of these models developed for a node had identical C++ code for both deployment on the device and simulation, except that the MQTTBroker was replaced with an actual MQTT Broker, and the ADC model and MQTTClient models were slightly modified.

For the ADC model, the internal transition function causes the hardware to poll the analog-to-digital channels during deployment on the hardware, whereas, for simulation, this model simply assigns a pseudo-random value to its state variable. For the MQTTClient model, the output port was removed, and in the output function, a call was made to an MQTT library to provide communication using this protocol.

To hold state variables and exchange messages through the input/output ports, the models used C++ primitive data types, such as int and double, the Standard Template Library (STL), such as std::map and std::vector, as well as two user-defined types. The two user-defined types, which were named ADCMessage and MQTTPacket, were used to store objects exchanged between the Wrapper model and the Base model, and the Base model and the MQTTClient, respectively. These types were defined using C++ template classes. 

For example, the type ADCMessage was defined as Model 6:
**Model 6** C++ Class for ADCMessagetemplate<class T> class ADCMessage { private: std::string originator; std::string deviceType;  T data; public: ADCMessage(std::string originator, std::string deviceType, T data): originator(originator), deviceType(deviceType), data(data) {}  T getData() const {return this->data;} void setData(T data) {this->data = data;}  void setRaw(std::string raw) {this->raw = raw;} std::string getRaw() const {return this->raw;}  void setOrigin(std::string originator) {this->originator = originator;}    std::string getOrigin() const {return this->originator;}   std::string getDeviceType() const {return this->deviceType;} } The template class could then be instantiated using a code such as: ADCMessage<double> msg(“24.3”, “temperature_sensors”, “sensor_1”);

Hence, the objects of this template class can hold data alongside information about the origin of the data.

The MQTTPacket model was defined similarly to ADCMessage, with the difference that its objects hold additional information about the topic to which the data should be published and the desired QoS level.

### 3.3. Verification of the Models via Simulation 

**Figure 4:**
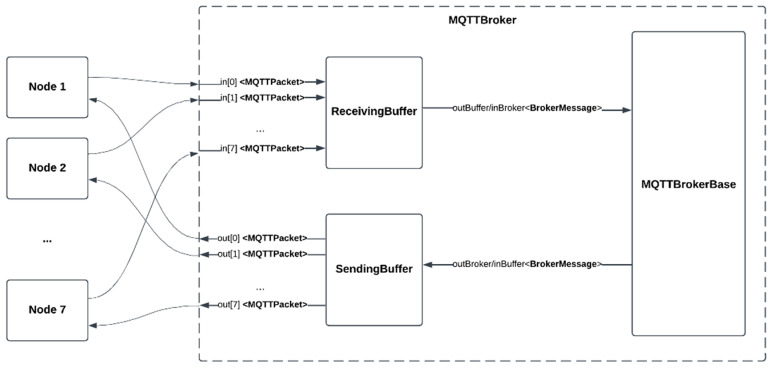
The coupling of IoT nodes to the message broker for simulation.

In what follows, we will discuss three case studies featuring seven nodes connected to the MQTTBroker model, with each node equipped with two redundant sensors. In this setup, the nodes periodically poll their sensors and transmit their readings to all other nodes. For all simulation runs, the feasible range of the sensor readings was set to be ±1 unit. Furthermore, the polling rate of the sensors attached to the nodes was set to 1 s, and 10 rounds of message exchange were performed between the nodes, causing the nodes to converge to common values. The number of message exchanges was set to 10 rounds, which would result in nodes having values that are suitably close to each other after performing message exchanges. To achieve even closer values between nodes, additional rounds of message exchange could be performed. Figure 4 shows the complete simulation setup.

**Figure 5:**
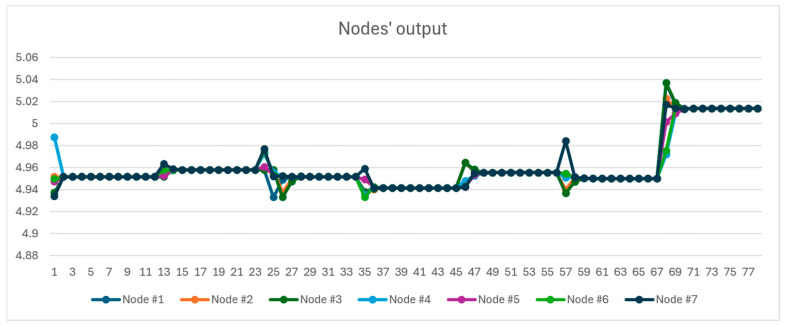
Nodes’ values in each iteration of message exchange in simulation #1.

The value that the sensor of each node reported was normally distributed with a mean of 5 and a standard deviation of 0.4. Figure 5 shows the output each node reported to the other nodes in different iterations of message exchange. As the values show, between each sensor polling, all nodes converge to common values, which were around the mean value of 5.

**Figure 6:**
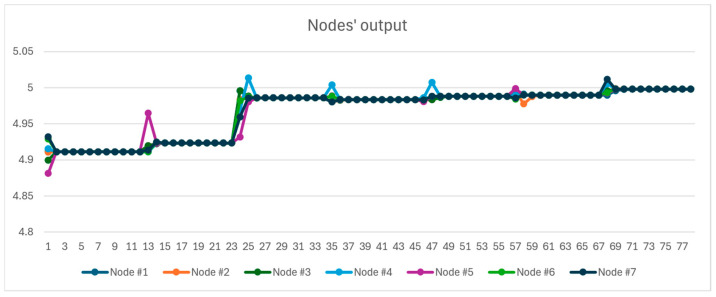
Nodes’ values in each iteration of message exchange in simulation #2.

In the second simulation run, as with the previous case, seven nodes were assumed to be in a situation in an environment, each with two redundant sensors. Contrary to the previous case, two nodes out of the seven nodes were assumed to be faulty and reported values normally distributed with a mean of 20 and standard deviations of 0.4. Figure 6 shows the output of each node in different timestamps. As the figure depicts, the value of the faulty nodes is discarded in each node, and each node’s output values, like the previous run, are closer to the mean value of 5.

## 4. Case Study—A Smart Home Application

In Section 3, we described an architecture based on the Brooks–Iyengar algorithm to achieve inexact agreement and sensor fusion in a network of sensors. The applicability of this architecture was demonstrated in a simulation setup consisting of multiple nodes, each with a set of redundant sensors. In this section, we will apply this approach to a home automation application as a case study.

A home automation setup consists of a set of sensors that read environmental variables, such as brightness and temperature, alongside a set of actuators that adjust environmental attributes. In this context, the environmental attributes are controlled based on sensor readings and user preferences, such as the desired room temperature set by the user. This setup, also termed a “smart home”, aims to enhance the energy efficiency, comfort, and security of its occupants. Home automation is one of the possible applications of IoT technology, as it integrates various devices and systems to enable interconnected and intelligent control of a home environment.

In our home automation case study, we have a network of “nodes”, which are microcontrollers equipped with either sensors or actuators. For the microcontroller, we chose ESP32 boards. The ESP32 is a series of system-on-a-chip microcontrollers with various capabilities, such as a built-in WiFi module and Bluetooth, making it suitable for IoT applications. Communication between the nodes is handled using the MQTT protocol.

For this study, a series of DEVS models were developed for both “sensor nodes”, i.e., ESP32 microcontrollers with sensors attached to them, as well as “actuator nodes” that have actuators connected to them. These models were first simulated, and after verifying their correct behavior, they were flashed onto the ESP32 microcontrollers for their operation. This method was made possible by using a DEVS simulator, which supported the execution of the models using both an event-based clock and a real-time clock. With the event-based clock, the simulator finds the next event to be executed in the hierarchy of the DEVS models and advances the simulation time instantaneously by that offset, allowing for rapid simulation. In contrast, when using a real-time clock, the models execute in real-time, making it suitable for deployment on hardware.

**Figure 7:**
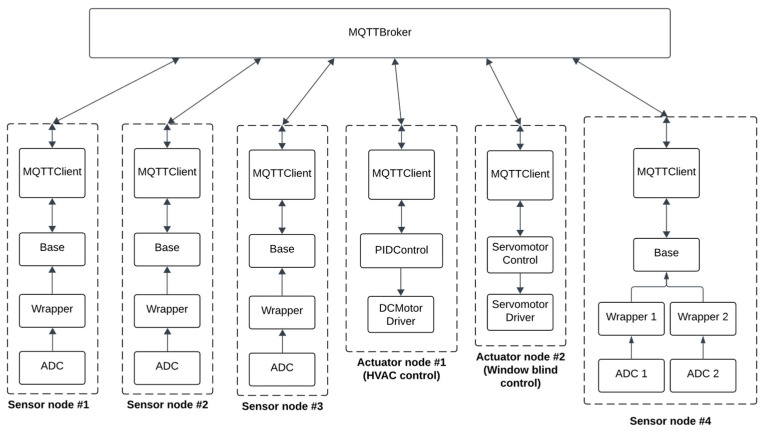
The architecture of the case study.

Figure 7 shows the complete architecture of the case study, comprising DEVS models executed on six nodes. As the figure shows, the case study consists of four sensor nodes and two actuator nodes. Three temperature sensors are connected to sensor nodes #1, #2, and #3, respectively. After capturing the environment’s temperature, these sensor nodes broadcast the values to each other and reach an inexact agreement via their Base model. Sensor node #4 is connected to two CO_2_ sensors, where the Base model fuses the CO_2_ readings and publishes them to the “/CO_2_/” topic. The fused values are published to the broker solely to monitor the CO_2_ level in the room. Finally, the two actuator nodes control a DC motor and a servo motor, which operate the HVAC system and a smart window blind, respectively.

In what follows, we will describe the DEVS models developed for the sensor nodes and actuator nodes.

### 4.1. Sensor Node Model

The DEVS models that run on a sensor node are those described for a “node” in Section 3. These models include the ADC model, Wrapper, Base, and MQTTClient. As described in Section 3, the ADC models poll the sensors connected to the board and send the data to the Wrapper model, which is then augmented with additional fields. The Base model is responsible for performing sensor fusion and ensuring inexact agreement between devices within the network. Lastly, the MQTTClient sends and receives data from the Base model.

We start by simulating these models, after which they are flashed on ESP32 boards equipped with two types of sensors. The first type was the “Grove Temperature Sensor V1.2” by Seeed Studio (https://wiki.seeedstudio.com/Grove-Temperature_Sensor_V1.2, accessed on 26 November 2024), a temperature sensor, and the second type, the “MH-Z19” Winsen Electronics Technology (https://www.winsen-sensor.com/sensors/co2-sensor/mh-z19c.html, accessed on 26 November 2024), was a CO_2_ sensor.

**Figure 8:**
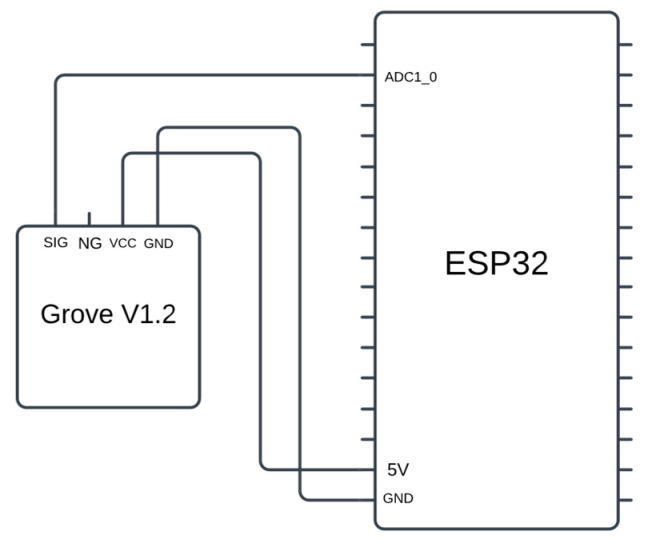
The electrical connection between ESP32 and Grove Temperature sensor.

The V1.2 temperature sensor we used has four pins: GND, VCC, SIG, and NG. The GND and VCC pins were connected to the GND and VCC pins of the ESP32 board, respectively. The SIG pin was connected to an ADC pin on the board. The electrical connection between the ESP32 board and the temperature sensor is depicted in Figure 8. This configuration enables the code running in the board’s ADC model to periodically poll the sensor and read and discretize the values it reported.

**Figure 9:**
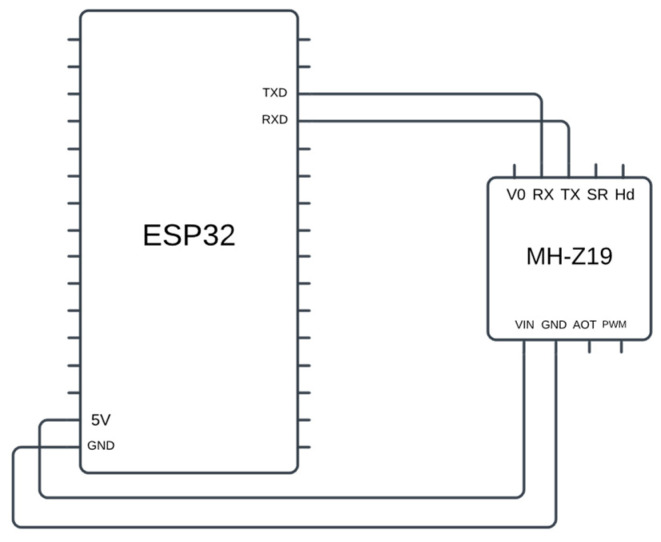
The electrical connection between ESP32 and MH-Z19 sensor.

The MH-Z19 CO_2_ sensor that we used, on the other hand, uses the UART to communicate with the board. The UART is a hardware peripheral that provides asynchronous communication between two devices using two cross-connected pins. Therefore, as shown in Figure 9, the RX and TX pins of the sensor were connected to the TX and RX pins of the ESP32 board, respectively. The other two pins of the CO_2_ sensor, labeled VCC and GND, were connected to the 5V power supply and the ground port of the microcontroller. These two pins provide electrical power to the sensor for its operation. Following this configuration, the code for the ADC model was also modified to allow communication using the UART peripheral. The first modification was calling the UART client API provided by ESP-IDF and setting the baud rate to 9600 according to the datasheet of the sensor. Next, a C++ function was written that ran concurrently with the main thread of the program, sending requests to the sensor to capture environmental values. These requests were 8-bit data following the format depicted in Table 1. This function receives a pointer to a function and runs it concurrently with the main thread of the program.

The response that the sensor sent back to the ESP32 was received by polling the sensor in a separate thread and a separate C++ function. These data followed the structure shown in Table 2.

The data received in each polling were parsed in the ADC model, and the “low level concentration” and “high level concentration” values were extracted. These two values were then used in Equation (1) to obtain the CO_2_ level of the environment.

(1) $${CO}_{2}\_level=high\_level\times 256+low\_level$$

After capturing the sensor values, the ADC model’s data are forwarded to the Wrapper model, the Base model, and the MQTTClient model, which then broadcasts the data to other clients within the network. As mentioned in Section 3, the Base model also receives data from other sensor nodes within the network and performs inexact agreement and sensor fusion.

### 4.2. HVAC Control Node

**Figure 10:**
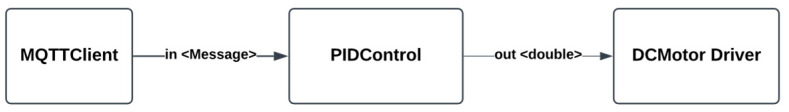
DEVS models for HVAC control node.

The HVAC control node is an ESP32 microcontroller connected to actuators that perform the heating, ventilation, and air conditioning (HVAC) of a room. The models developed for this node are depicted in Figure 10. The MQTTClient model receives sensor readings from “sensor nodes” within the network and forwards them to the PIDControl model. The PIDControl model receives the readings and controls the actuators based on these values and a “setpoint” defined for the model. In this context, the setpoint could refer to the desired temperature of a room, such as for a heating system.

The MQTTClient, in addition to sensor data, can also receive setpoint values from users. This is achieved by the MQTTClient model, which subscribes to MQTT topics where users can publish their desired setpoints. The new setpoint values received by the MQTTClient are also forwarded to the PIDControl model. The PIDControl model differentiates between sensor readings and new setpoint values by performing a lookup on the metadata received alongside the message.

In our case study, the actuator performing HVAC is a DC motor connected to the board. Choosing DC motors allowed us to demonstrate the different actuating commands generated by the HVAC control node by observing the motor’s speed. In this setup, the PIDControl model controls the motor’s speed by outputting a control signal proportional to the desired speed. The PIDControl model generates this signal based on the PID principle. The output is then sent to the DCMotorDriver model, which receives a value proportional to the desired speed and generates a pulse-width-modulated (PWM) signal to control the motor’s speed. A PWM signal is an electrical signal with varying gaps between its pulses; smaller gaps correspond to higher electrical power, resulting in higher motor speed. Since the input to the DCMotorDriver is proportional to the motor’s speed, this model generates a PWM signal only for non-negative input values. Therefore, a negative input results in a PWM signal with a zero duty cycle.

The PIDControl model runs on the HVAC control node as a discretized PID controller with an anti-windup method. This model is defined as follows (Model 7):
**Model 7** PIDControl ModelPIDControl = <S, X, Y, δint, δext, λ, ta> S = { phase ∈ {active, passive}, outputSignal, setpoint} X = {in} Y = {out} δext (s,e,x) = { switch(inData.type)  case newSetPoint:   setPoint = inData.data    case sensorData:     double e = reference − inData; outputSignal = outputSignal + Kp × (e − prev_error) + (Ki) × Ts × e + (Kd/Ts)(e – 2 × prev_error + prev_prev_error) if(outputSignal > limMax)//Anti wind-up    outputSignal = limMax     else if(outputSignal < limMin)       outputSignal = limMin prev_prev_error = prev_error; prev_error = e; phase = active } δint (s) = {  phase = passive; } λ (s) = {   if(phase == active)   out = outputSignal } ta(active) = 0 ta(passive) = infinity

The MotorDriver model had two states: passive and active. Whenever the model received the desired speed of the motor from the PIDControl model, it transitioned to active, generated the PWM signal on the pin of the board, and then changed state back to passive.

### 4.3. Smart Window Blind

A smart window blind is a window blind that can be controlled remotely. In our case study, we implemented a smart blind device using an ESP32 controller connected to a servomotor. A servomotor is an electromechanical actuator capable of precisely adjusting its shaft to the desired angle. Internally, servomotors use feedback received from their “encoders”—sensors that can sense the shaft’s position and velocity—to control the shaft to the appropriate position. For the case study, the “SG90” servomotor was used. This device receives commands as a pulse-width-modulated (PWM) signal with a period of 20 ms (50 Hz) and a varied duty cycle between 1 and 2 ms. A duty cycle of 1 ms denotes the −90-degree position, while a duty cycle of 2 ms indicates the 90-degree angle.

**Figure 11:**
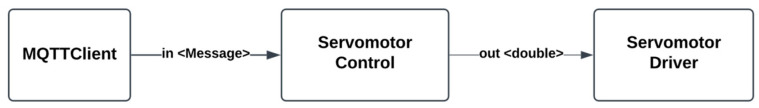
DEVS models for the smart blind device.

Figure 11 depicts the models that were developed for the smart blind device.

Similar to previous models, the MQTTClient provides connectivity for the devices. The ServomotorControl model receives the desired angle of the shaft and produces different shaft angles, starting from the current angle of the shaft to the desired angle over time. This behavior ensures that the servomotor will be set at a specific angle with a smooth transition. Lastly, the ServomotorDriver receives the angle and generates a PWM signal that drives the servomotor.

The DEVS specification of the ServomotorControl is as follows (Model 8):
**Model 8** ServomotorControl ModelServomotorControl = <S, X, Y, δint, δext, λ, ta> S = {phase ∈ {rotating, passive}, currentAngle, targetAngle} X = {in} Y = {out} δext (s,e,x) = { targetAngle = inData phase = rotating } δint (s) = { if currentAngle < targetAngle:  currentAngle = currentAngle + 1 else if currentAngle > targetAngle:  currentAngle = currentAngle - 1 else  phase = passive } λ (s) = {   if(phase == rotating)  outData = currentAngle } ta(rotating) = 0 ta(passive) = infinity

### 4.4. Simulation and Deployment of the Models

The DEVS models for the sensor node were simulated in Section 3; therefore, their description will be omitted in this section. To simulate the PIDControl model, we needed a plant model that captured the characteristics of the real system under control. For our case study, this required a model of the DC motor as well as a complex model that represented the diffusion of heat in the specific room. Developing such models, particularly the one for heat diffusion, was challenging since parameters such as the locations of windows, doors, and the movement of people in the room could significantly affect the model’s behavior. To validate the PID control principle used in the PIDControl model via simulation, a generic second-order system with arbitrary coefficients and a time-delayed input was used instead.

The plant was modeled as a second-order system, as many physical systems are governed by nth-order differential equations. For instance, the authors in [16] used a second-order system to describe a gas fire heater. After selecting a model for the plant, the PIDControl model was validated by observing the plant’s output when subjected to input from the PIDControl model. Given that the plant’s output approached the desired set point within a reasonable time, it was concluded that the PID control was producing valid results. The equation for the plant was as follows:

(2) x(t−10)=16y″(t)+y′(t)+y(t)

**Figure 12:**
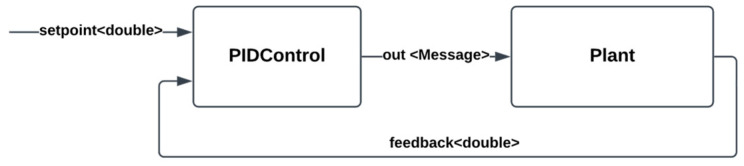
The models and their coupling to simulate the PIDControl model.

Here, x is the input and y are the output of the plant. The input is delayed by 10 s to capture the delay that is present between the action the actuator performs on the plant and the output that the sensor receives. In the case study, this delay is caused by the delay in CO_2_ or heat diffusion. The PIDControl DEVS model was coupled with the DEVS model of the plant, as depicted in Figure 12. The plant receives input from PIDControl and solves the second-order system numerically using Euler’s method.

**Figure 13:**
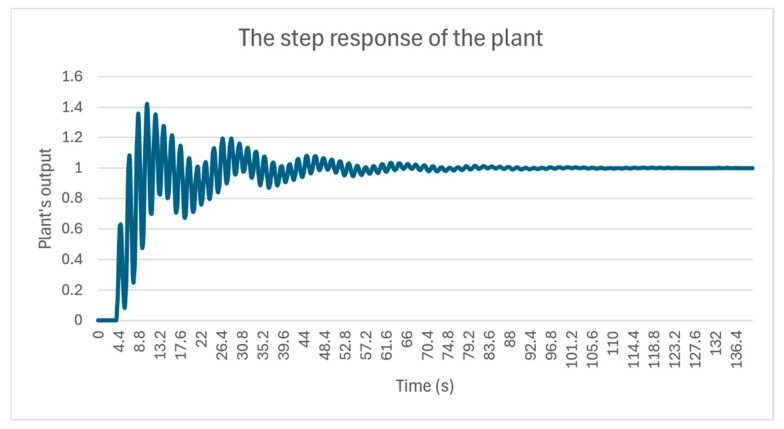
The plant’s output subjected to the input from the PIDControl model.

After running multiple simulation instances and performing parameter tuning, the PID control constants for the PIDControl were determined to be 0.58 and 0.71 for the proportional and integral terms, respectively, and 0 for the derivative term. These values provide a suitable compromise between a rapid response of the plant and maintaining reasonable oscillation. The output of the plant to a step input for the PID coefficients is depicted in Figure 13.

**Figure 14:**
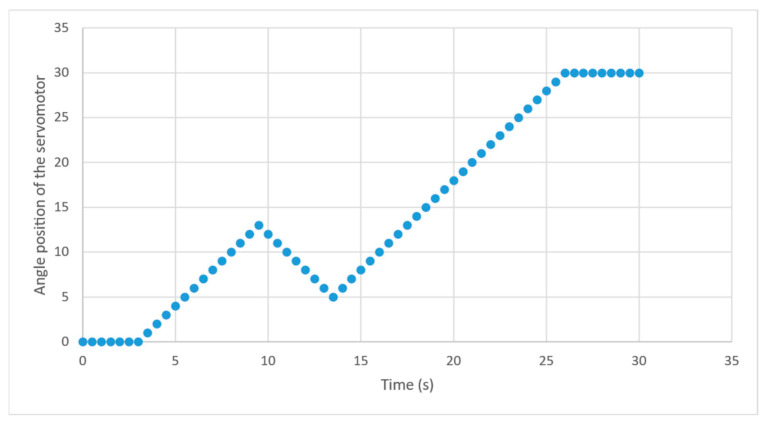
Angle of the servomotor under simulation in different timestamps.

Simulation of the ServomotorControl for the smart blind device was carried out by observing the output it generated for different desired angles, including the edge cases. Figure 14 shows the output of the ServomotorControl subject to various inputs applied at different timestamps. The servomotor initially had an angle of 0 and received a command to reach an angle of 40 at timestamp 3. At timestamp 9, the servomotor received the target angle of 0, and at timestamp 13, it received the target value of 30. The servomotor changed its angle from that time until it reached the target value, where it stayed at that angle.

**Figure 15:**
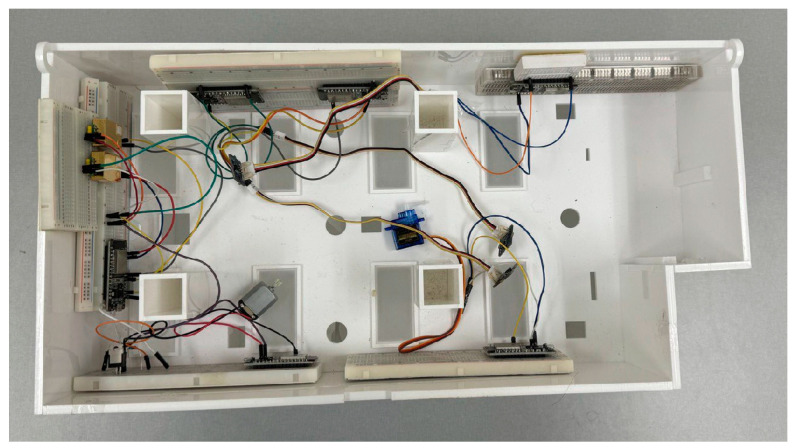
Deployment of the sensors on the VSim scale model room.

After simulation, the models were flashed to different ESP32 devices and deployed in a 3D model at a scale of room 3222 VS, as depicted in Figure 15.

This room is a replica at a scale of room 3222 VS, in the VSim building at Carleton University. This model has been laser-cut from a BIM model of the room available from the School of Architecture and CIMSLab. Three temperature sensors and two CO_2_ sensors were connected to four ESP32 boards. The remaining two ESP32 devices were used as follows: one controlled a servomotor, while the other managed a DC motor via a transistor.

After deploying the devices, the sensors were subjected to different inputs, and the behavior of the actuators was observed. The results from applying varied inputs confirmed the simulation results. For instance, applying a heat source to only one sensor did not affect the speed at which the DC motor (i.e., the cooler) operated. This was because this node was considered “faulty” by the Brooks–Iyengar algorithm and was excluded from broadcasting to other nodes.

#### 4.4.1. Simulation

The simulation of the models in Figure 7 was carried out by providing input to the ADC models at different timestamps and observing the behavior of the other models in the architecture. In Cadmium, this was achieved using the “IEStream” model, which reads an input file consisting of timestamps and data and then outputs the data at the specified timestamps through its output port.

Table 3 shows the data provided to the ADC models. The first column, which represents the timestamp range, specifies the period during which the ADC model outputs a value. The feasible range of the readings was set to ±2 units.

**Figure 16:**
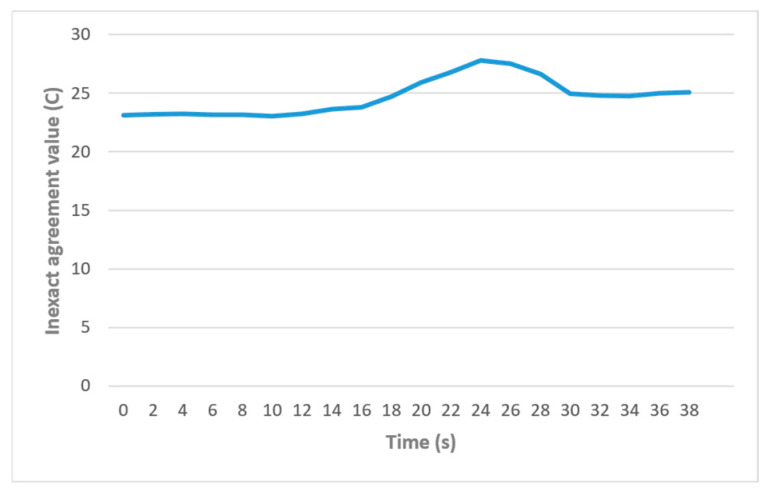
Inexact agreement in the case study in different iterations.

The inputs given to the temperature ADC models in Table 3 (columns 1–3) are values that were exchanged between the three nodes to achieve an inexact agreement. Figure 16 shows the result of this inexact agreement between the nodes. As the figure shows, the nodes agree on values near 20 degrees until 12s in the simulation (corresponding to the timestamp range 10–12 in Table 3). After this point, the values start to increase, reaching 28 degrees as the nodes report higher values. The agreed value then drops after 24 s, corresponding to the timestamp range 22–24 in the table.

**Figure 17:**
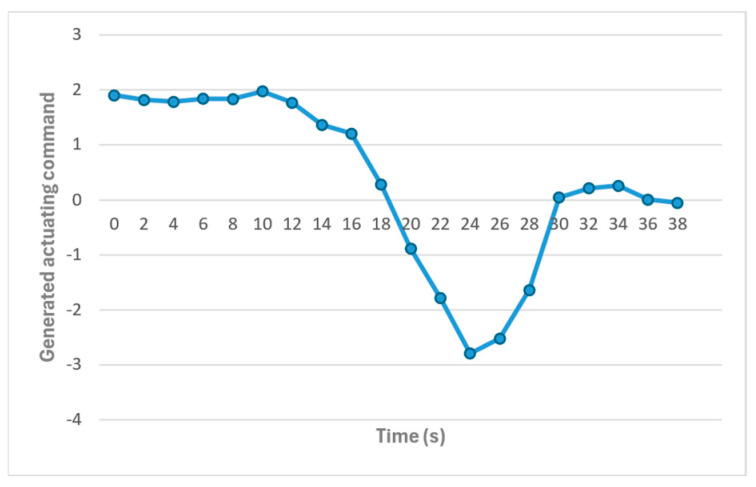
Actuating command generated by the PIDControl model.

After achieving approximate agreement between each ADC poll, the results were sent to the node with the DC motor to perform proper HVAC control using the PID control model. The model responsible for performing PID control, known as the PIDControl model, produced the appropriate control signal for the DCMotorDriver model, which, in turn, logged the electrical signal it generated to a file for the simulation. The setpoint for the PIDControl model was chosen to be 25 degrees. Figure 17 shows the output of this model for the temperature values after reaching the inexact agreement. The figure shows that PIDControl, by following PID control principles, generates positive actuating signals until the point where the value of the nodes surpasses the setpoint of 25 degrees Celsius. After this timestamp, a negative signal is generated for sensor values above 25 degrees until the temperature decreases to 25 degrees, at which point the actuating signal becomes zero.

**Figure 18:**
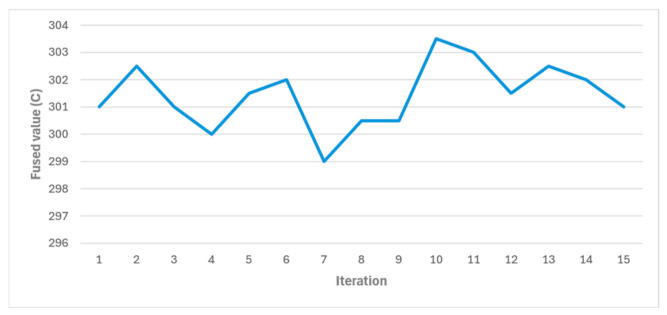
The fused CO_2_ sensor readings in the Base model.

The inputs provided to ADCs of the node with CO_2_ sensors were fused together in the Base model and transmitted from the device. Since these were the only CO_2_ sensors connected to the nodes within the network, there was no inexact agreement between nodes. Figure 18 shows the fused data from these sensors. The figure shows that the fused value is around 300, which was anticipated since the CO_2_ sensor readings were both close to this value. The algorithm simply “fused” the readings from these sensors without exchanging messages, resulting in an inexact agreement.

#### 4.4.2. Deployment

In this section, we will discuss the changes made to the DEVS models to make them suitable for deployment on the hardware. The first modification involved updating the ADC model to poll the hardware ADC on the board instead of using an input file to generate data. In the ESP32 microcontroller and the ESP-IDF compiler suite, this was achieved using the APIs provided by the esp_adc/adc_oneshot.h header file. After including this header and the necessary code for ADC configuration, the ADC channel of the board was read using the adc_oneshot_read function call.

In the simulation, the MQTTClient model simply transmitted the messages it received from the Base model to the MQTT broker model, which then forwarded them to other nodes based on subscription relationships. For deployment, the MQTTClient model was modified to execute API calls that communicate with the actual MQTT protocol, utilizing functions provided by mqtt_client.h in ESP-IDF.

Lastly, the ServomotorDriver and MotorDriver models were also modified for deployment. In the simulation, these models logged output to a file. For deployment, both models were updated to generate appropriate PWM signals to control the servomotor via API calls provided by driver/mcpwm_prelude.h, specifying the proper duty cycle of the PWM.

After modification, the models were flashed onto the devices and connected via a serial USB cable to a computer to log the results in real-time.

**Figure 19:**
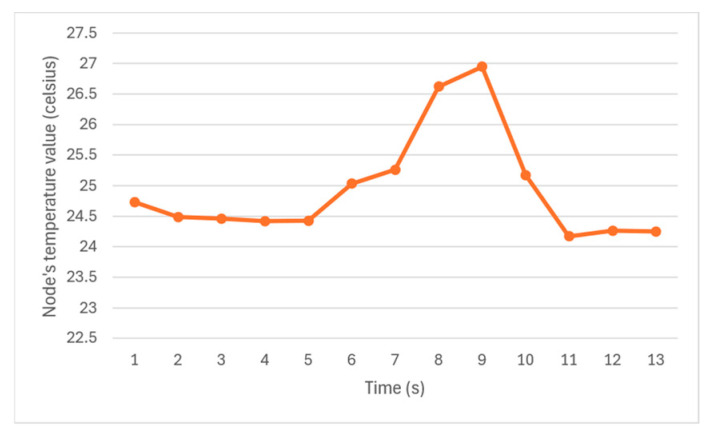
The temperature the nodes agree on in different timestamps.

Figure 19 shows the temperatures reported by a node after achieving inexact agreement with other nodes. The figure shows that the temperature readings initially start around 24.5 degrees and increase after 5 s, reaching a peak of 27 degrees at 9 s. This behavior results from the heat applied to two out of three sensors at 5 s timestamp until 9 s. After the 9 s, a low temperature was applied to the sensors, which caused the temperature to drop and return to approximately 24 degrees from 11 s onward. The figure indicates that inexact agreement occurs among the three sensors when the number of faulty nodes is below the threshold defined by the Brooks–Iyengar algorithm. The value from the faulty sensor was simply discarded in this test scenario, which was as desired.

**Figure 20:**
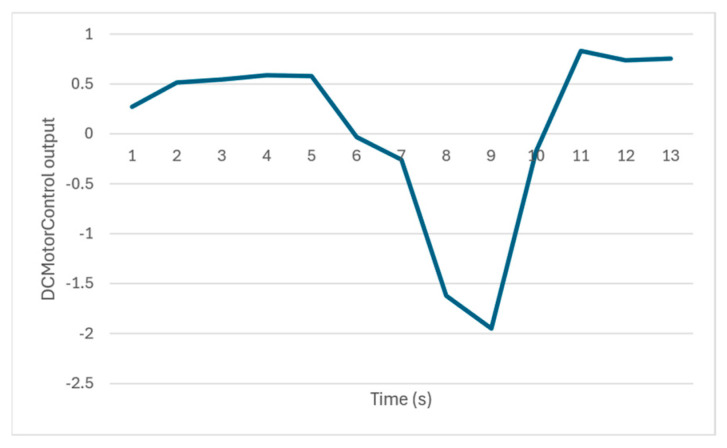
The actuating command the PIDControl model generates.

The output of the PIDControl model, which is based on the temperature data reported by the nodes, is depicted in Figure 20. The actuating command generated by the model is initially positive, around 0.5, until 5 s, when it decreases, crosses zero, and reaches a minimum value of −2 at 9 s. This minimum value corresponds to the peak value reported by the nodes. The model’s output then increases, crosses zero again, and stabilizes around 0.7. These values align with the trend of the temperature values in the environment depicted in Figure 19 and their difference from the reference value of 25.

The servomotor control was independent of any sensor readings. The output of the model, subject to different desired target angles, was identical to that shown in Figure 14.

## 5. Conclusions

The Internet of Things (IoT), as a paradigm encompassing interconnected sensors and actuators, has been applied to various domains, including home automation, health monitoring, and transportation. The use of IoT in these technologies leads to the automation of tasks, enables real-time monitoring of assets and operations, and improves efficiency while facilitating the management of systems and resources.

The complexity of these systems, which stem from the use of various processing units, sensor types, communication technologies, and the environments in which they are deployed, leads to challenges related to interoperability, human interaction, and system heterogeneity. These issues complicate the development of such systems and the verification of the correctness of the components when they are working together.

Model-based approaches have been proposed in the literature to overcome these challenges. These approaches involve using high-level semantics to create models that are executed on the devices. These models can be expressed in various formats, such as those based on languages with formal or informal semantics or in graphical or textual formats. Depending on the approach, these models can then be used for both verification purposes as well as being transformed into executable code that runs on IoT devices.

In this work, we applied DEVS formalism as a model-based approach to develop such applications. For this purpose, we developed a series of models, each responsible for a specific functional aspect of the device. 

These functionalities include polling one or multiple sensors, transmitting the data to other nodes, and applying a modified Brooks–Iyengar algorithm to the sensor data. These models were then combined to form the complete model intended to be executed on each device.

We simulated a network of nodes running these models. The simulation results showed that the nodes could successfully combine sensor readings from their redundant sensors into a single value and exchange messages with other nodes to “agree” on common values, provided that the number of faulty nodes is below the tolerated threshold. This is achieved by each node collecting readings from neighboring nodes, applying the Brooks–Iyengar algorithm, and exchanging the results with neighboring nodes. Multiple iterations of this process cause the nodes’ values to converge closer to a common value. Various simulation scenarios with different configurations were conducted, and the results aligned with the desired behavior of the models.

To demonstrate the applicability of the developed models in practice, a home automation application was created consisting of multiple sensors and actuators. The sensors included CO_2_ and temperature sensors. The actuators were a DC motor and a servo, representing an HVAC unit and a smart blind, respectively. In this setup, the DC motor was intended to be controlled by the sensor outputs, whereas the servomotor was intended to be controlled solely based on user input.

## Figures and Tables

**Table 1:** The structure of the UART message transmitted to the CO_2_ sensor.

Send Command								
Byte0	Byte1	Byte2	Byte3	Byte4	Byte5	Byte6	Byte7	Byte8
Starting byte	Sensor No.	Command	-	-	-	-	-	Check Value
0xFF	0x01	0x86	0x00	0x00	0x00	0x00	0x00	0x79

**Table 2:** The structure of the UART message received from the CO_2_ sensor.

Response								
Byte0	Byte1	Byte2	Byte3	Byte4	Byte5	Byte6	Byte7	Byte8
Starting byte	Command	High level concentration	Low level concentration	-	-	-	-	Check Value

**Table 3:** The input given to temperature sensors of the case study.

**Timestamp** **Range**	**ADC Node #1** **(Temperature Sensor)**	**ADC Node #2** **(Temperature Sensor)**	**ADC 1 Node #3** **(Temperature Sensor)**	**ADC 2 Node #2** **(CO_2_ Sensor)**	**ADC 3 Node #2** **(CO_2_ Sensor)**
0–2	20	20.2	20	300	302
2–4	20	19.8	20.2	302	303
4–6	20	20	20.1	301	301
6–8	20	20.5	20	300	300
8–10	20	20.3	20.5	301	302
10–12	21	21	21	303	301
12–14	23.6	23.6	23.6	299	299
14–16	23.7	23.7	23.7	300	301
16–18	23.9	23.9	23.9	300	301
18–20	24	24	24	310	312
20–22	24.2	24.2	24.2	305	301
22–24	25	25	25	301	302
24–26	25.2	25.3	25.6	304	301
26–28	25	25.3	25.5	301	303
28–30	25.2	25.1	25.1	301	301

## Data Availability

Data are contained within the article.

## References

[1] Nguyen X.T., Tran H.T., Baraki H., Geihs K. FRASAD: A Framework for Model-Driven IoT Application Development. Proceedings of the 2015 IEEE 2nd World Forum on Internet of Things (WF-IoT).

[2] Doddapaneni K., Ever E., Gemikonakli O., Malavolta I., Mostarda L., Muccini H. A Model-Driven Engineering Framework for Architecting and Analysing Wireless Sensor Networks. Proceedings of the 2012 Third International Workshop on Software Engineering for Sensor Network Applications (SESENA).

[3] Wainer G.A., Glinsky E., MacSween P. (2005). A Model-Driven Technique for Development of Embedded Systems Based on the DEVS Formalism. Model-Driven Software Development.

[4] Zeigler B.P., Praehofer H., Kim T.G. (2000). Theory of Modeling and Simulation.

[5] Arslan S., Ozkaya M., Kardas G. (2023). Modeling Languages for Internet of Things (IoT) Applications: A Comparative Analysis Study. Mathematics.

[6] Murata T. (1989). Petri Nets: Properties, Analysis and Applications. Proc. IEEE.

[7] Pnueli A. The Temporal Logic of Programs. Proceedings of the 18th Annual Symposium on Foundations of Computer Science (sfcs 1977).

[8] Harrand N., Fleurey F., Morin B., Husa K.E. ThingML: A Language and Code Generation Framework for Heterogeneous Targets. Proceedings of the ACM/IEEE 19th International Conference on Model Driven Engineering Languages and Systems.

[9] Thramboulidis K., Christoulakis F. (2016). UML4IoT—A UML-Based Approach to Exploit IoT in Cyber-Physical Manufacturing Systems. Comput. Ind..

[10] Costa B., Pires P.F., Delicato F.C., Li W., Zomaya A.Y. Design and Analysis of IoT Applications: A Model-Driven Approach. Proceedings of the 2016 IEEE 14th Intl Conf on Dependable, Autonomic and Secure Computing, 14th Intl Conf on Pervasive Intelligence and Computing, 2nd Intl Conf on Big Data Intelligence and Computing and Cyber Science and Technology Congress (DASC/PiCom/DataCom/CyberSciTech).

[11] Lamport L., Shostak R., Pease M. (2019). The Byzantine Generals Problem. Concurrency: The Works of Leslie Lamport.

[12] Mahaney S.R., Schneider F.B. Inexact Agreement: Accuracy, Precision, and Graceful Degradation. Proceedings of the Fourth Annual ACM Symposium on Principles of Distributed Computing.

[13] Nakamura E.F., Loureiro A.A., Frery A.C. (2007). Information Fusion for Wireless Sensor Networks: Methods, Models, and Classifications. ACM Comput. Surv. (CSUR).

[14] Marzullo K. (1990). Tolerating Failures of Continuous-Valued Sensors. ACM Trans. Comput. Syst. (TOCS).

[15] Brooks R.R., Iyengar S.S. (1996). Robust Distributed Computing and Sensing Algorithm. Computer.

[16] Sameer R., Badgwell T.A. (2000). Robust disturbance rejection for FIR systems with bounded parameters. IFAC Proc. Vol..

